# When suppressor effects may reflect multicollinearity artifacts: a cautionary tale from multiple object tracking and clay pigeon shooting performance

**DOI:** 10.3389/fpsyg.2026.1908043

**Published:** 2026-09-14

**Authors:** Kai Jin, Pingze Gao, Shuo Han, Xupeng Zhang

**Affiliations:** 1 Department of Physical Education, Southeast University, Nanjing, China; 2 Liaoning Provincial Sports Bureau, Shenyang, China; 3 School of Information and Software Engineering, University of Electronic Science and Technology of China, Chengdu, China; 4 Electric Power Research Institute, State Grid Jiangsu Electric Power Co., Ltd., Nanjing, China; 5 Department of Physical Education, Nanjing University of Aeronautics and Astronautics, Nanjing, China

**Keywords:** clay pigeon shooting, multicollinearity, multiple object tracking, predictive validity, statistical artifact, Suppressor effect, variance inflation factor

## Abstract

**Objective:**

To evaluate whether multiple object tracking (MOT) predicts clay pigeon shooting performance, and to illustrate how multicollinearity between demographic covariates can mimic suppressor effects.

**Methods:**

Twenty shooters completed a MOT task (2–6 targets). Zero-order and partial correlations, hierarchical regression, LOOCV, VIF diagnostics, and power analysis were computed. Two specifications were compared: Specification A (age + training years, multicollinear) and Specification B (age only, VIF < 2.5).

**Results:**

Age and training years were near-perfectly correlated (r = 0.928). Under Specification A r=0.928;VIF=11.7 , MOT-5 and MOT-6 appeared as nominally significant partial correlates (r_partial ≈ 0.49, *p* < 0.03, uncorrected), a classical suppression pattern. Under Specification B, these effects attenuated to non-significance (*p* > 0.13). The training coefficient reversed sign (B = −2.12 despite r = +0.65), consistent with multicollinearity distortion. Power analysis indicated N ≈ 68 would be required to detect the residual effect (f^2^ = 0.15), though this estimate is illustrative given the small sample. AUC=0.660f2=0.12.

**Conclusion:**

The apparent suppressor effect is consistent with multicollinearity between age and training years rather than genuine prediction, though the small sample (*N* = 20) precludes definitive conclusions. Routine VIF, bootstrap stability checks, and power analysis are recommended to avoid artifacts in small-sample sports cognition research.

## 1 Introduction

Clay pigeon shooting demands exceptional dynamic visual attention: athletes must track targets moving at speeds up to 110 km/h and complete the full sensorimotor sequence within approximately 0.5–0.8 s of target launch (Causer et al., 2010; Abernethy and Neal, 1999), making the sport a natural testbed for theories of visual attention in high-performance environments (Meyerhoff et al., 2017; Huff et al., 2012; Causer et al., 2011).

The Multiple Object Tracking (MOT) paradigm, introduced by Pylyshyn and Storm (1988), is the most widely used laboratory assay for dynamic visual attention. A substantial literature has established that athletes outperform non-athletes on MOT tasks, particularly under high cognitive load (4–6 simultaneously tracked targets) (Liu et al., 2024; Jin et al., 2023; Qiu et al., 2018; Voss et al., 2010; Huttermann et al., 2014). However, this literature has focused almost exclusively on group differences, expert versus novice, athlete versus non-athlete, without testing whether MOT performance predicts real-world competitive outcomes within trained populations. The latter criterion, predictive validity, is the standard required for any measure proposed as a talent identification tool (Lidor et al., 2009; Williams and Reilly, 2000).

When evaluating cognitive predictors of athletic performance, demographic covariates, particularly age and training experience, present both a statistical necessity and a statistical hazard. These variables are typically strong predictors of both the cognitive measure and the competitive outcome, creating the conditions for confounding. Failure to control for them yields biased estimates; however, when age and training experience are themselves highly collinear, as they inevitably are in athletic samples where older athletes have accumulated more training years, entering both as simultaneous covariates can produce its own set of artifacts. Specifically, the combination of near-perfect collinearity among covariates and partial correlation analysis can generate patterns that mimic classical statistical suppression (Conger, 1974; MacKinnon et al., 2000; Paulhus et al., 2004), wherein a variable appears unrelated to an outcome in zero-order correlation but emerges as a “significant” predictor after covariate control.

Distinguishing genuine suppression from a multicollinearity artifact requires explicit diagnostic procedures that are rarely reported in the sports cognition literature. Variance inflation factor (VIF) diagnostics can identify problematic collinearity (O’Brien, 2007), but VIF alone does not resolve the tension between two competing interpretations: (a) the collinearity distorts coefficient estimates, producing inflated significance estimates (the multicollinearity interpretation); or (b) both covariates carry independent information, and omitting one introduces omitted variable bias that underestimates the predictor’s true effect (the confound-control interpretation). One approach (Wooldridge, 2019) is to examine coefficient stability: sign reversals relative to zero-order relationships may indicate multicollinearity distortion, whereas directionally consistent coefficients would be more consistent with proper confound control.

The present study uses MOT performance and competition scores from a sample of clay pigeon shooters to illustrate this diagnostic sequence. We show that (a) entering both age and training years as simultaneous covariates produces a pattern consistent with a substantive suppressor effect, with MOT-5 and MOT-6 emerging as nominally significant partial correlates; (b) VIF diagnostics reveal severe multicollinearity (r = 0.928, VIF = 11.7 and 10.8); (c) the training years coefficient undergoes a sign reversal (negative in the regression despite a positive zero-order r = +0.650 with scores), indicating that multicollinearity distortion rather than omitted variable bias is the more parsimonious account; (d) when the model is respectified with only age as a covariate, the apparent suppressor effect attenuates substantially; and (e) power analysis indicates that a sample of approximately 68 participants would be required to detect the residual effect (f^2^ = 0.15) at 80% power, an illustrative estimate derived from the same small sample. The contribution of this paper is the demonstration that routine diagnostic procedures, VIF, coefficient stability checks, and specification sensitivity analysis, can distinguish substantive findings from statistical artifacts in a research context where the two are easily confused.

## 2 Methods

### 2.1 Participants

All participants (or, for those under 18 years of age, a parent or legal guardian) provided written informed consent, and minor participants provided written assent.

The sample comprised 9 skeet shooters and 11 trap shooters (12 male, 8 female). Mean age was 19.9 ± 3.9 years (range: 14–26) and mean training experience was 6.8 ± 2.9 years (range: 2–12). Competition scores, averaged across each athlete’s recorded competitions, ranged from 95.0 to 115.2 (M = 106.2, SD = 6.3). Skeet (*n* = 9) and trap (*n* = 11) shooters did not differ significantly in mean competition score (104.8 ± 5.5 vs. 107.4 ± 6.9, *p* = 0.372) or in MOT accuracy (MOT-5: 0.600 vs. 0.636, *p* = 0.669; MOT-6: 0.411 vs. 0.409, *p* = 0.985), although the small sample provides limited power to detect discipline differences, and the absence of a significant difference should not be interpreted as evidence of equivalence. Female participants had slightly higher mean scores than males (107.1 vs. 105.7), though this difference was not statistically significant (point-biserial r = +0.117, *p* = 0.624). Critically, age and training years were near-perfectly correlated (r = 0.928, *p* < 0.001; shared variance = 86.0%), reflecting the natural confounding in this population: older athletes have, by definition, accumulated more years of specialized training. No participants reported color blindness, recent ocular surgery, or use of attention-affecting medications. All testing was completed within a single session lasting approximately 90 min.

### 2.2 Multiple object tracking task

The MOT task was programmed in Matlab R2016a using Psychtoolbox-3 (Brainard, 1997) and presented on a 14-inch laptop (1920 × 1,080 pixels, 60 Hz refresh rate). Viewing distance was 60 cm (chin rest), ambient illumination 300 lx. Twelve white circles (1.2 cm diameter, ~1.15° visual angle) moved randomly at 5°/s, a speed selected to match prior MOT research with athletic populations (Jin et al., 2023; Qiu et al., 2018) while remaining within the range where tracking is feasible for all participants. During the 1,200-ms indexing phase, target circles turned blue and flashed three times. The 10-s tracking phase was followed by an untimed response period. The formal experiment comprised five blocks (2, 3, 4, 5, and 6 targets; 30 trials each) with block order counterbalanced via Latin square. An additional exploratory condition presented 3 targets at increased speeds of 10°/s and 15°/s (MOT-10°/s and MOT-15°/s, respectively). Seven MOT variables were derived as mean tracking accuracy (proportion of correctly identified targets) for each condition; the 10°/s and 15°/s conditions are analysed descriptively in Section 3.7.

### 2.3 Competition score

Competition scores were obtained from official national-level match records (National Shooting Championships, National Shooting Champions Series, National Junior Shooting Championships, and the National Games). For each athlete, the score is the mean of that athlete’s scores across all recorded competitions (each individual competition scored as total targets hit, maximum 125 for both skeet and trap); because scores are averaged over a variable number of competitions (range 1–10 per athlete), the outcome takes non-integer values.

### 2.4 Statistical analysis

All analyses were conducted in Python 3.12 (scikit-learn 1.9, SciPy 1.17, statsmodels 0.14). Analysis code and a de-identified dataset are provided as Supplementary material to facilitate reproducibility. Significance threshold: α = 0.05, two-tailed. Exact *p*-values are reported unless *p* < 0.001. Given the exploratory nature of the study and the number of MOT variables examined (seven), readers should note that the partial-correlation *p*-values reported in Section 3.3 are uncorrected for multiple comparisons; with seven MOT variables, a Bonferroni-corrected threshold would be α = 0.007.

Zero-order and partial correlations. Pearson correlations were computed between each MOT variable and competition score, with bootstrap 95% confidence intervals (10,000 resamples). Partial correlations were computed in two specifications: Specification A, controlling for age and training years simultaneously; Specification B, controlling for age only. Throughout, Specification A refers to age + training years and Specification B to age only; gender is reported descriptively (Section 3.1) and is not included as a covariate in either specification.

Multicollinearity diagnostics. Variance inflation factors were computed for all predictors. Following conventional guidelines, VIF > 10 was considered indicative of severe multicollinearity requiring model scrutiny (O’Brien, 2007).

Hierarchical regression and coefficient stability analysis. Competition score was regressed on demographic covariates in Step 1, with MOT-5 and MOT-6 (identified *post hoc* as the two variables showing the suppression pattern) added in Step 2. Both specifications were analysed. Because MOT-5 and MOT-6 were selected after inspecting the partial-correlation results, the subsequent hierarchical regression and cross-validation analyses are illustrative diagnostics rather than confirmatory tests. To adjudicate between multicollinearity distortion and omitted variable bias, we examined the sign and magnitude of the training years coefficient under Specification A relative to its zero-order correlation with competition scores. A coefficient sign reversal (i.e., a negative regression coefficient despite a positive zero-order r) is compatible with multicollinearity distortion but can also occur under genuine suppression; it is therefore interpreted together with the other diagnostics rather than as conclusive on its own (Wooldridge, 2019). Bootstrap standard errors were obtained by case resampling (5,000 resamples, fixed seed) of the regression coefficient under each specification. Effect size was quantified as Cohen’s f^2^. Power analysis was conducted using the non-central F distribution (α = 0.05, target power = 0.80).

Cross-validation. Leave-one-out cross-validation (LOOCV) was performed for three models: MOT alone (MOT-5 + MOT-6), age alone, and age + MOT-5 + MOT-6. Cross-validated R^2^ and RMSE are reported.

Exploratory speed analysis. Trial-level data (N = 141) were analysed descriptively. Because trials are nested within participants and therefore non-independent, no inferential statistics are reported for this analysis. Results are presented solely to motivate future independent-sample investigations.

## 3 Results

### 3.1 Descriptive statistics and zero-order correlations

Descriptive statistics and zero-order correlations are presented in Table 1. Tracking accuracy decreased monotonically with target load (MOT-2: 0.965 ± 0.059; MOT-6: 0.410 ± 0.231). No MOT variable correlated significantly with competition scores (|r| = 0.04–0.33, all *p* ≥ 0.160, uncorrected for multiple comparisons; Figure 1). Age was a strong predictor (r = +0.741, *p* < 0.001; R^2^ = 0.549), as was training years (r = +0.650, *p* = 0.002). Gender showed a small, non-significant correlation with scores (point-biserial r = +0.117, *p* = 0.624), with female participants scoring slightly higher on average (107.1 vs. 105.7). Notably, despite this weak zero-order relationship, adding gender to a model with age alone increased R^2^ by 7.4 percentage points (from 0.549 to 0.623), a modest suppressor-like increment that, with N = 20, most plausibly reflects sampling variability rather than a population effect—a parallel cautionary illustration of how unstable covariate effects can be in small samples. The critical diagnostic finding is the near-perfect correlation between age and training years (r = 0.928, *p* < 0.001; shared variance = 86.0%), which motivates all subsequent specification comparisons. The observations-per-predictor ratios were 5.0:1 for Specification A (20 observations, 4 predictors) and 6.7:1 for Specification B (20 observations, 3 predictors); both ratios fall below the commonly recommended minimum of 10:1 for stable regression estimates, warranting caution in interpreting individual coefficient magnitudes.

**Table 1:** Descriptive statistics and zero-order Pearson correlations with competition score (*N* = 20).

Variable	M	SD	Min	Max	r	p
MOT-2	0.965	0.059	0.80	1.00	−0.208	0.380
MOT-3	0.860	0.127	0.50	1.00	+0.039	0.872
MOT-4	0.770	0.134	0.50	0.90	−0.227	0.336
MOT-5	0.620	0.182	0.20	0.90	+0.132	0.579
MOT-6	0.410	0.231	0.10	0.90	−0.106	0.657
MOT-10°/s	0.710	0.112	0.50	1.00	−0.059	0.804
MOT-15°/s	0.615	0.184	0.10	0.90	+0.327	0.160
Age	19.90	3.89	14	26	+0.741	<0.001
Training years	6.78	2.86	2	12	+0.650	0.002
Gender					+0.117	0.624

*r*(Age, Training Years) = 0.928 (*p* < 0.001); *r*(Age, Score) = 0.741; *r*(Training Years, Score) = 0.650.

**Figure 1:**
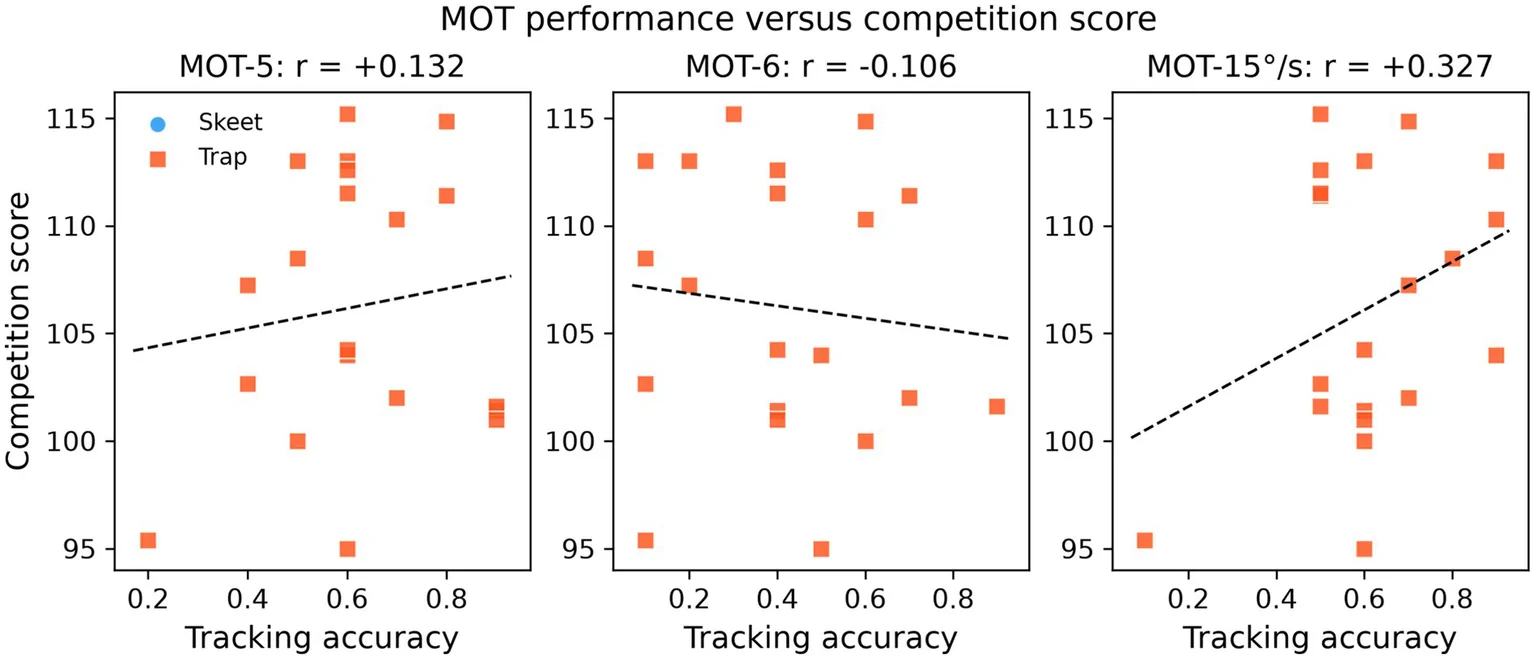
MOT performance (MOT-5, MOT-6, MOT-15°/s) versus competition score. Dashed lines indicate OLS regression fits. All zero-order relationships are non-significant (all *p* ≥ 0.05).

### 3.2 Multicollinearity diagnostics

Variance inflation factors are reported in Table 2. Under Specification A, age (VIF = 11.7) and training years (VIF = 10.8) exhibited severe multicollinearity, substantially exceeding the VIF > 10 threshold (O’Brien, 2007), whereas MOT-5 and MOT-6 showed acceptable values (2.3–2.5). When training years was removed (Specification B), all remaining VIF values fell below 2.5, indicating that the collinearity was confined to the age–training pair.

**Table 2:** Variance inflation factors.

Predictor	VIF (Spec. A: Age + Training)	VIF (Spec. B: Age only)
Age	11.7	1.3
Training years	10.8	
MOT-5	2.3	2.0
MOT-6	2.5	2.4

VIF > 5: Problematic collinearity; VIF > 10: Severe collinearity.

### 3.3 Partial correlations: specification a versus specification B

Table 3 and Figure 2 present the critical comparison. Under Specification A (controlling for both age and training years), MOT-5 and MOT-6 showed nominally significant positive partial correlations: r_partial = +0.495 (*p* = 0.027) and r_partial = +0.488 (*p* = 0.029), a pattern consistent with classical statistical suppression (Conger, 1974; MacKinnon et al., 2000), complete with a sign reversal for MOT-6 (from r = −0.106 to r_partial = +0.488). These *p*-values are uncorrected for multiple comparisons across seven MOT variables; with Bonferroni correction (α = 0.007), neither would reach significance. Under Specification B (controlling for age only), these effects attenuated substantially: MOT-5 r_partial = +0.318 (*p* = 0.172); MOT-6 r_partial = +0.345 (*p* = 0.137). Specification B serves as a pragmatic sensitivity model for assessing the influence of multicollinearity rather than the unequivocally correct specification; this issue is examined further in Sections 3.4 and 3.9.

**Table 3:** Zero-order and partial correlations.

Variable	r (zero-order)	*p*	r_partial (Spec. A)	p	r_partial (Spec. B)	*p*
MOT-2	−0.208	0.380	−0.036	0.880	−0.038	0.872
MOT-3	+0.039	0.872	+0.132	0.580	+0.149	0.530
MOT-4	−0.227	0.336	−0.226	0.338	−0.233	0.324
MOT-5	+0.132	0.579	+0.495	0.027	+0.318	0.172
MOT-6	−0.106	0.657	+0.488	0.029	+0.345	0.137
MOT-10°/s	−0.059	0.804	+0.148	0.533	+0.070	0.771
MOT-15°/s	+0.327	0.160	+0.074	0.756	+0.025	0.917

Spec. A: Controlling for age + training (multicollinear). Spec. B: Controlling for age only (VIF < 2.5 for all predictors).

**Figure 2:**
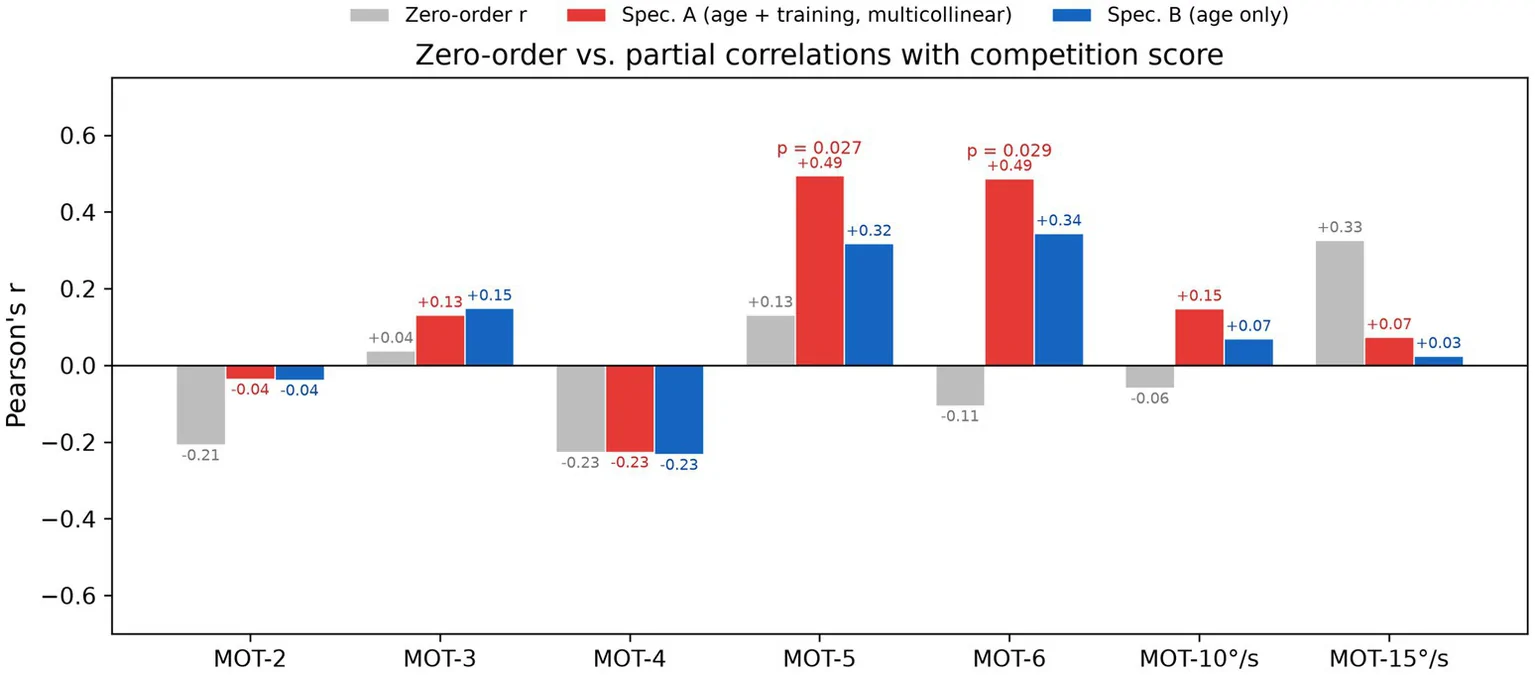
Zero-order correlations versus partial correlations under Specification A (age + training, multicollinear) and Specification B (age only). Red bars: Specification A (multicollinear). Blue bars: Specification B (age only); All *p* > 0.13.

### 3.4 Hierarchical regression and coefficient stability

Hierarchical regression results are presented in Table 4. Under Specification A, adding MOT-5 and MOT-6 yielded ΔR^2^ = 0.133 (*F*(2,15) = 3.26, *p* = 0.067, f^2^ = 0.43). Under Specification B, ΔR^2^ fell to 0.059 (*F*(2,16) = 1.19, *p* = 0.328, f^2^ = 0.15).

**Table 4:** Hierarchical regression of competition score on demographic and MOT predictors, with bootstrap standard errors (5,000 resamples).

	Spec. A (Age + Training)	SE (Boot)	Spec. B (age only)	SE (Boot)
Step 1 R^2^	0.559	-	0.549	-
Step 2 R^2^	0.693	-	0.608	-
ΔR^2^	0.133	-	0.059	-
F-change	*F*(2,15) = 3.26	-	*F*(2,16) = 1.19	-
p (ΔR^2^)	0.067	-	0.328	-
f^2^	0.43	-	0.15	-
Age	+2.87	0.77	+1.34	0.35
Training	−2.12	1.10		
MOT-5	+8.89	11.46	+3.48	11.72
MOT-6	+6.90	8.31	+4.85	8.47

Bootstrap standard errors based on 5,000 resamples. See Section 3.4 for interpretation of coefficient stability metrics.

The coefficient stability analysis provides the key diagnostic evidence. Under Specification A Step 2, the training years coefficient was B = −2.12, negative and opposite in sign to its zero-order correlation with scores (r = +0.650). This sign reversal is compatible with multicollinearity distortion: when two near-perfectly collinear covariates are entered simultaneously, their coefficients can take on implausible values of opposite sign as the estimation procedure attempts to partition the shared variance (Wooldridge, 2019) (see Figure 3 for the full correlation matrix). The age coefficient under Specification A was B = +2.87, substantially larger than its bivariate regression coefficient (B ≈ 1.20 from a simple regression of Score on Age alone), further indicating coefficient inflation. By contrast, under Specification B, the age coefficient was directionally consistent with its zero-order relationship, and the MOT-5 coefficient (B = +3.48) was directionally consistent with its weak positive zero-order correlation (r = +0.132), though its extremely wide confidence interval reflects the coefficient instability that small samples permit.

**Figure 3:**
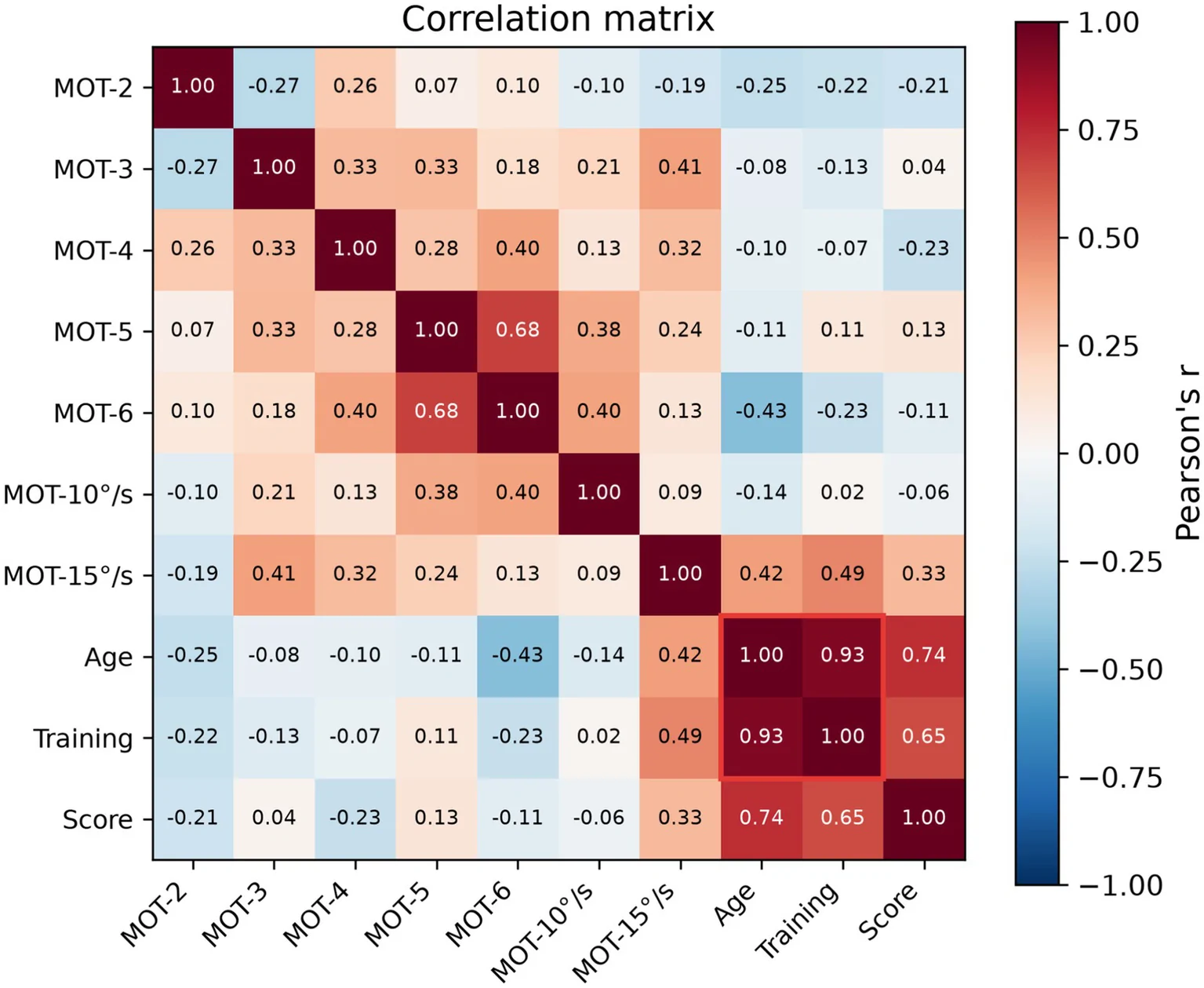
Correlation matrix. The near-perfect age–training correlation (*r* = 0.928, top-right), the dominant predictor status of age (*r* = 0.741 with score), and the uniformly weak MOT–score correlations.

LOOCV cross-validation further corroborates this conclusion (Table 5). Under Specification B, age alone achieved R^2^_cv = 0.446; adding MOT-5 and MOT-6 yielded R^2^_cv = 0.456 (ΔR^2^_cv = +0.010), a negligible increment (Figure 4). The MOT-only model performed substantially worse than the mean baseline (R^2^_cv = −0.376), further indicating that high-load MOT does not contribute positively to out-of-sample prediction. Because MOT-5 and MOT-6 were selected on the full sample, feature selection was not nested within the cross-validation loop; the LOOCV estimates should therefore be interpreted as a further diagnostic of the within-sample suppression pattern rather than as evidence of out-of-sample predictive generalizability.

**Table 5:** Leave-one-out cross-validation results (Specification B).

Model (Spec. B)	R^2^_cv	RMSE
MOT-5 + MOT-6 only	−0.376	7.22
Age only	0.446	4.58
Age + MOT-5 + MOT-6	0.456	4.54

**Figure 4:**
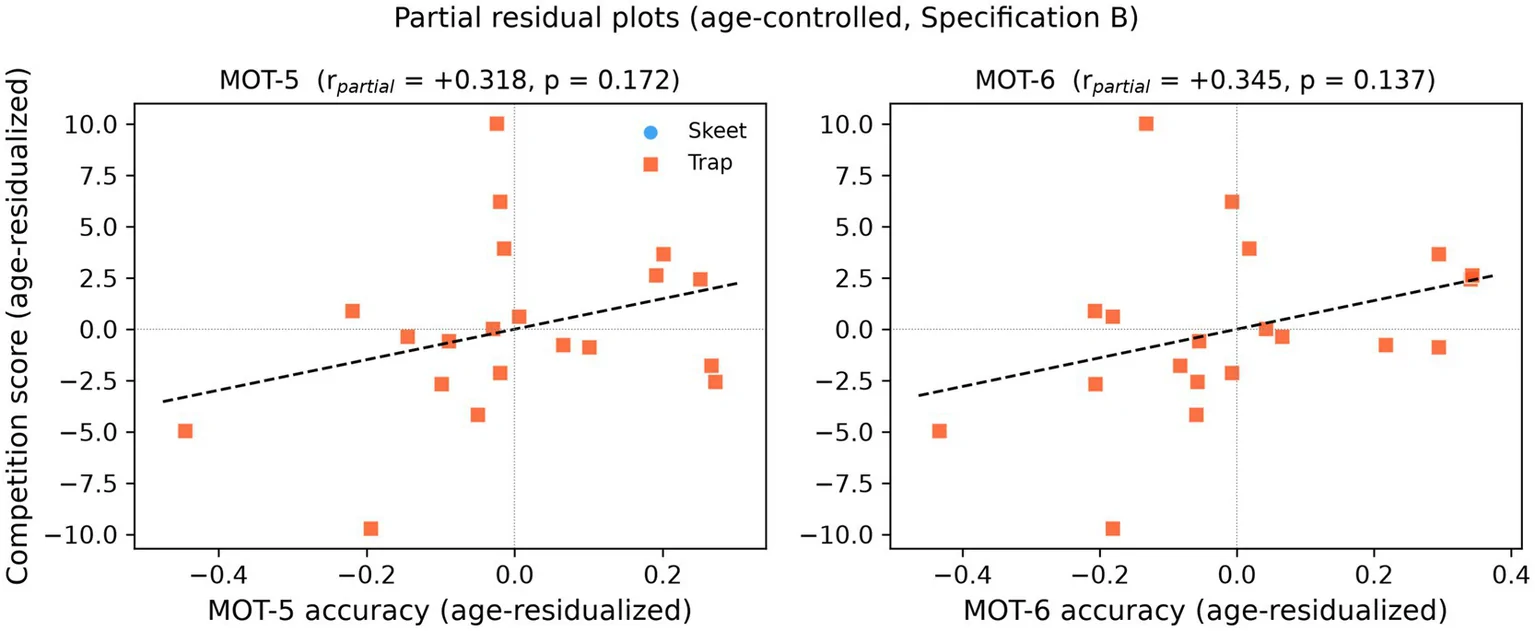
Partial residual plots (age-controlled) for MOT-5 and MOT-6 under Specification B. The positive slopes are attenuated and non-significant (*p* = 0.172 and *p* = 0.137).

Several diagnostics from Table 4 merit emphasis. First, the Age coefficient standard error under Specification A (SE_boot = 0.77) was 2.21 times larger than under Specification B (SE_boot = 0.35), directly quantifying the variance inflation produced by multicollinearity. Second, the Training coefficient under Specification A underwent a sign reversal (B = −2.12 despite r_zero-order = +0.650), and its bootstrap 95% CI spanned from −4.74 to −0.42. Third, the MOT-5 and MOT-6 coefficients were highly unstable: under Specification B their bootstrap 95% CIs spanned zero (MOT-5: −6.03 to +42.45; MOT-6: −22.94 to +12.90), with a positive sign in only 72 and 77% of bootstrap resamples, respectively. These bootstrap intervals transparently communicate the uncertainty inherent in coefficient estimates from small samples.

### 3.5 Power analysis

Under Specification B, the incremental contribution of MOT-5 and MOT-6 beyond age alone was f^2^ = 0.15, computed from the observed ΔR^2^ — a sample estimate, not an *a priori* planning value, that happens to coincide with Cohen’s conventional “medium” benchmark (Cohen, 1988). The power analysis therefore addresses the F-test for ΔR^2^ (the incremental contribution of the two MOT predictors after controlling for age) rather than the overall regression model. With α = 0.05, target power = 0.80, 2 tested predictors and 1 control predictor, approximately 68 participants would be required to detect an incremental effect of this magnitude. Because f^2^ = 0.15 was itself estimated from this small and unstable sample, the N ≈ 68 figure is best regarded as an illustrative planning estimate rather than a precise requirement. The present sample (*N* = 20) provides approximately 27% power, insufficient to distinguish between a genuinely null effect and a small effect that the study is underpowered to detect.

### 3.6 Exploratory: speed-dependent patterns

An exploratory analysis of trial-level data (*N* = 141) examined MOT–score correlations across speed and target-load conditions (Figure 5). Because trials are nested within participants and therefore non-independent, no inferential statistics are reported; these descriptive patterns are presented in the Supplementary material solely to guide future independent-sample investigations.

**Figure 5:**
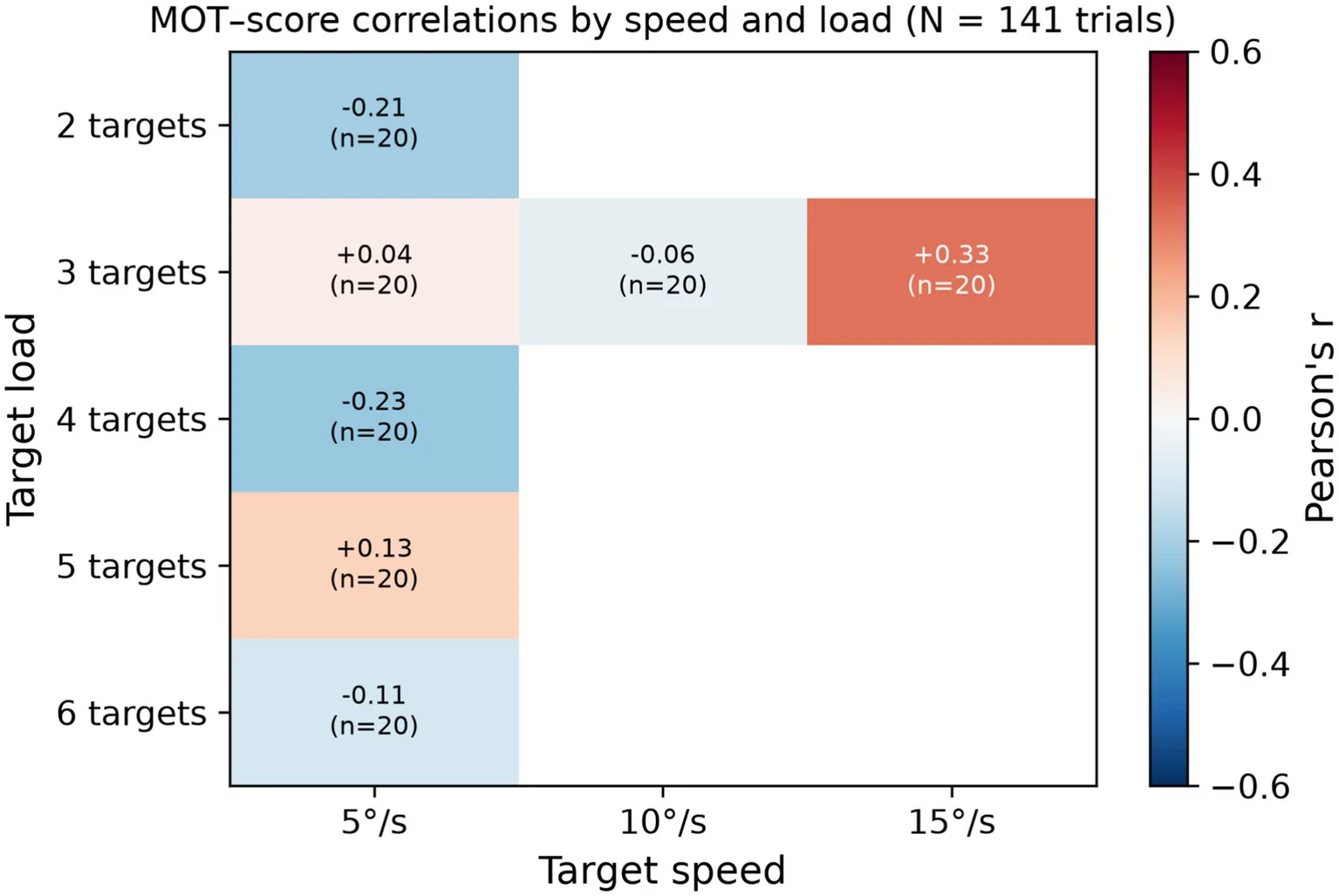
Descriptive speed × target load patterns in MOT–score correlations (*N* = 141 non-independent trials). See Supplementary material for details. No inferential statistics are reported.

### 3.7 Sensitivity analysis: multicollinearity versus omitted variable bias

A critical interpretive question is whether Specification B (age only) is genuinely preferable to Specification A (age + training), or whether removing training years introduces omitted variable bias that could mask a true MOT–performance relationship, should one exist in the population. The foregoing analyses provide convergent evidence favoring the multicollinearity-distortion account. VIF diagnostics (§3.2) confirmed severe collinearity between age and training (VIF > 10). Hierarchical regression (§3.4) revealed that the training-years coefficient under Specification A reversed sign (B = −2.12) relative to its strong positive zero-order correlation with scores (r = +0.650), a pattern compatible with multicollinearity distortion (Wooldridge, 2019) and difficult to reconcile with omitted variable bias. Bootstrap standard errors (Table 4) showed that the Age coefficient SE was 2.21 times larger under Specification A than under Specification B, directly quantifying the variance inflation produced by collinearity, and both MOT-5 and MOT-6 coefficients had bootstrap 95% CIs spanning zero under Specification B. LOOCV (§3.4) further showed a negligible out-of-sample increment (ΔR^2^_cv = +0.010) when MOT predictors were added to the age-only model. This convergent pattern, spanning VIF, coefficient stability, SE inflation, bootstrap instability, and cross-validation, indicates that multicollinearity distortion, rather than omitted variable bias or genuine suppression, is the more parsimonious account for this dataset, though the limited sample size precludes a definitive ruling.

## 4 Discussion

This study began with a straightforward question, does MOT performance predict competition scores in clay pigeon shooters? and arrived at a methodological finding that we believe is more instructive than a simple answer. When we entered both age and training years as covariates, a seemingly natural analytical choice, we obtained a pattern that textbook descriptions would identify as a classical suppressor effect: MOT-5 and MOT-6 were unrelated to scores at the zero-order level but emerged as significant positive correlates after covariate control, complete with a sign reversal for MOT-6. This pattern, had we stopped there, might have been interpreted as evidence that high-load MOT predicts competition performance but is masked by demographic confounding.

However, three diagnostic checks converged to suggest that this pattern more likely reflects a multicollinearity artifact than a genuine suppressor effect. First, VIF diagnostics revealed severe collinearity between age and training years (r = 0.928, VIF > 10). Second, the training years coefficient in the fully specified model was negative (B = −2.12) despite a strong positive zero-order correlation with scores (r = +0.650), a sign reversal compatible with multicollinearity distortion (Wooldridge, 2019). Third, when the model was respectified with only age as a covariate, the apparent suppressor effect attenuated to non-significance: MOT-5 r_partial fell from +0.495 (*p* = 0.027) to +0.318 (*p* = 0.172); MOT-6 from +0.488 (*p* = 0.029) to +0.345 (*p* = 0.137). Power analysis indicated that a sample of approximately 68 participants would be required to detect the residual effect (f^2^ = 0.15) at 80% power—an illustrative estimate—underscoring that the present study lacks the statistical power to rule out a small genuine effect.

The coefficient sign reversal under Specification A merits particular emphasis because, combined with the other diagnostics, it helps distinguish multicollinearity distortion from omitted variable bias. The concern that removing training years introduces omitted variable bias is legitimate in principle: if training experience carries information about competition performance that is independent of age, removing it could underestimate the MOT effect. However, the negative coefficient for training years (B = −2.12) is implausible, it would imply that, holding age constant, each additional year of training reduces competition scores by more than 2 points. This implausibility reflects the statistical reality that, when age and training share 86% of their variance, the regression cannot meaningfully separate their independent contributions. The “unique” training years effect that remains after partialling out age is not a valid measure of training-specific experience; it is residual noise with no coherent interpretation. In such cases, econometric best practice recommends dropping one of the collinear variables (Wooldridge, 2019), and age, as the stronger zero-order predictor (r = 0.741 vs. 0.650), is the natural choice.

The broader implication is not that MOT is irrelevant to shooting performance, our power analysis leaves open the possibility of a small genuine effect that the present study cannot resolve, but rather that standard analytical workflows in small-sample sports cognition research may be insufficiently guarded against multicollinearity artifacts when demographic covariates are inherently confounded. Furthermore, unmeasured individual-difference factors, such as video-game experience, which has been shown to modulate visuospatial cognitive performance (Feng et al., 2007), may further complicate the interpretation of cognitive-performance relationships in small samples. We recommend the seven practices detailed in Section 4.1. These practices add minimal computational burden and provide essential protection against interpreting artifacts as discoveries.

A further implication concerns the published MOT expertise literature. In typical expert–novice comparisons, the expert group is older and has accumulated more sport-specific training than the novice group, precisely the demographic confounding documented here. Recent meta-analytic evidence indicates that visual skill-performance associations are generally modest and heterogeneous across sports and study designs (Fleddermann et al., 2026). While many of these studies report controlling for age, few report VIF diagnostics or coefficient stability checks. The present findings raise the question of whether some published “expert advantages” in MOT performance might partially reflect unexamined demographic collinearity rather than genuine cognitive expertise effects. We do not suggest that the MOT expertise literature is unreliable; rather, we note that the diagnostic framework illustrated here could be productively applied to existing datasets to quantify the robustness of published findings to alternative model specifications. The fact that the present case initially produced a pattern consistent with a textbook suppressor effect, complete with sign reversal and nominally significant partial correlations, before diagnostic scrutiny suggested an alternative interpretation underscores the value of such re-examination. More broadly, evidence that MOT performance is trainable across the lifespan (Legault et al., 2013) and that MOT training can transfer to sport-specific decision-making (Romeas et al., 2016) suggests that MOT-based measures retain practical utility for talent development, provided that demographic confounds are properly modelled.

Several limitations should be noted. First, the sample size (N = 20) is the most significant constraint, yielding an observations-per-predictor ratio of 5.0:1 for Specification A and 6.7:1 for Specification B, both below the commonly recommended minimum of 10:1 for stable regression estimates (Wooldridge, 2019). The analytical scope, encompassing hierarchical regression, bootstrap resampling, LOOCV, and power analysis, is substantial relative to the available data, and results should be interpreted accordingly. While this sample size is typical for elite athletic populations, it provides only 27% power to detect f^2^ = 0.15. The confidence intervals around all reported effect sizes are correspondingly wide. Multi-site collaboration will likely be necessary to achieve the N ≈ 68 indicated by the power analysis. Second, the cross-sectional design precludes causal inference regarding the developmental trajectory of MOT and its relationship to competition performance. Third, the competition score was averaged over a variable number of competitions per athlete (range 1–10), so score reliability differs across participants and temporal stability was not assessed. Fourth, the coefficient stability argument for preferring Specification B, while grounded in standard econometric diagnostics (Wooldridge, 2019), is not a formal statistical test; it is a pattern of convergent evidence. Fifth, the near-perfect age–training correlation (r = 0.928) in this sample may reflect the specific recruitment context (athletes from structured training programmes who began specialized training at a consistent age) and may not generalize to samples with more heterogeneous training histories. Sixth, participants were recruited from a single provincial training base and one military sports school, both in eastern China, limiting generalizability to other competitive levels, shooting disciplines (e.g., double trap, sporting clays), geographic regions, and athlete populations. Seventh, the combination of skeet and trap athletes into a single sample, while justified by shared visual–attentional demands and co-training context, may nonetheless obscure discipline-specific differences in perceptual–motor requirements; the null discipline comparisons in Section 2.1 should not be read as evidence of equivalence, given the small sample. Eighth, the *p*-values for MOT-5 and MOT-6 partial correlations under Specification A were not corrected for multiple comparisons across seven MOT variables; with Bonferroni correction, neither would reach the adjusted threshold.

### 4.1 Practical recommendations for talent identification research

Based on the diagnostic framework illustrated in this case study, we offer the following concrete recommendations for researchers and practitioners evaluating cognitive predictors of athletic performance:

1. Compute and report VIF for all predictors in any regression model with multiple demographic covariates. If VIF > 5 for any covariate, flag it; if VIF > 10, consider model respecification. In the present case, VIF > 10 for both age and training years triggered model respectification.
2. When VIF is elevated, conduct a specification sensitivity analysis: compare the primary model against a reduced model retaining only the single strongest demographic predictor. Report both sets of partial correlations, hierarchical regression results, and LOOCV predictions side-by-side (as in Tables 3–5).
3. Examine coefficient stability through bootstrap resampling (minimum 1,000 resamples). Report bootstrap standard errors alongside point estimates, and verify that coefficients maintain directional consistency with their zero-order relationships. A sign reversal in a demographic covariate (e.g., a negative coefficient despite a positive zero-order r, as observed for Training in this study) is a warning flag requiring investigation.
4. Do not rely on coefficient sign reversal alone as evidence for or against any interpretation, because sign reversal is the defining feature of both genuine statistical suppression and multicollinearity distortion. Convergent evidence from VIF, SE inflation, and bootstrap stability is needed for confident diagnosis.
5. Conduct and report an explicit power analysis for the key hypothesis test (e.g., the F-test for ΔR^2^). In small samples typical of elite athletic research, effect sizes may be meaningfully large (f^2^ ≥ 0.15) without reaching statistical significance. Reporting the sample size required for 80% power (N ≈ 68 in the present case, an illustrative estimate) contextualises non-significant findings and guides future study design.
6. Report the observations-per-predictor ratio for each specification. In the present study, Specification A had 5.0 observations per predictor (20/4) and Specification B had 6.7 (20/3). Ratios below 10 warrant caution; ratios below 5 warrant explicit acknowledgment as a limitation, as the model approaches saturation and individual influential points can substantially affect coefficient estimates.
7. If the study involves athletes from structured talent development programmes where training onset age is relatively uniform, expect age and training years to be very highly correlated [e.g., r > 0.85, corresponding to VIF > 5 for the demographic covariates (O’Brien, 2007)]. Under these conditions, we recommend using age as the primary demographic covariate and reporting training years descriptively rather than as a simultaneous covariate.

These recommendations add modest computational burden, approximately ten lines of code in standard statistical software, and provide essential protection against interpreting multicollinearity artifacts as substantive discoveries.

## 5 Conclusion

This study provides convergent multimethod evidence that an apparent suppressor effect of multiple object tracking on clay pigeon shooting competition performance is consistent with multicollinearity between age and training years (r = 0.928, shared variance = 86.0%; VIF > 10; bootstrap SE inflation ratio = 2.21 × for Age). Five lines of evidence converge on this interpretation: (a) VIF diagnostics indicating severe collinearity; (b) a coefficient sign reversal for training years under the multicollinear specification (B = −2.12 despite r_zero-order = +0.650); (c) bootstrap SE inflation (Age SE_boot = 0.77 under Specification A vs. 0.35 under Specification B); (d) coefficient instability under both specifications (MOT-5 and MOT-6 bootstrap 95% CIs spanning zero under Specification B); and (e) attenuation of the apparent MOT effect to non-significance when the model was respectified with only age as a covariate (ΔR^2^_cv = +0.010). However, the small sample (N = 20) provides only 27% power to detect the residual effect (f^2^ = 0.15), and approximately 68 participants would be required for adequate power—an illustrative planning estimate. The findings therefore cannot rule out a small genuine predictive effect of high-load MOT on shooting performance. Practical implementation guidance for these diagnostic procedures is provided in Section 4.1.

## Data Availability

The original contributions presented in the study are included in the article/Supplementary material, further inquiries can be directed to the corresponding author/s.
