# Supplementary material for: When suppressor effects may reflect multicollinearity artifacts: a cautionary tale from multiple object tracking and clay pigeon shooting performance
Source: Front Psychol. 2026 Sep 14;17:1908043. doi: 10.3389/fpsyg.2026.1908043 (PMC13617100; doi:10.3389/fpsyg.2026.1908043)
Supplement: Supplementary file 1 [file Table_1.DOCX]

Supplementary Material

When Suppressor Effects May Reflect Multicollinearity Artifacts: A Cautionary Tale from Multiple Object Tracking and Clay Pigeon Shooting Performance

Kai Jin, Pingze Gao, Shuo Han, Xupeng Zhang

# S1. Exploratory Speed-Dependent Analysis

This supplement provides the detailed exploratory analysis of speed-dependent patterns in MOT–score correlations, referenced in Section 3.7 of the main manuscript.

## S1.1 Methods

In addition to the formal MOT experiment described in Section 2.2, an exploratory condition presented 3 targets at increased speeds of 10 and 15°/s. Trial-level data from all speed conditions (5, 10, and 15°/s) were pooled, yielding N = 141 data points across speed × target-load combinations. Because trials are nested within participants and therefore non-independent, no inferential statistics are computed; all results are descriptive and presented solely to guide future independent-sample investigations.

## S1.2 Results

Figure 5 in the main manuscript displays the descriptive correlation patterns between MOT accuracy and competition scores across speed × target-load conditions. Several descriptive trends were observed:

At the standard speed (5°/s), MOT–score correlations were uniformly weak across all target loads (2–6 targets), consistent with the zero-order correlations reported in Table 1.

At the increased speeds (10 and 15°/s), MOT-15°/s showed the numerically largest zero-order correlation with scores (r = +0.327, p = 0.160, uncorrected), though this did not reach significance and was not robust to covariate adjustment (Table 3); by contrast, MOT-10°/s showed a weak, non-significant association (r = −0.059). At the trial level, correlations across speed × target-load combinations were descriptively variable, but the small number of trials per condition and the non-independence of observations preclude meaningful interpretation of these fluctuations.

Across all speed conditions, no consistent pattern of increasing or decreasing MOT–score correlations with speed or target load emerged.

## S1.3 Interpretation and Caution

These descriptive patterns should be interpreted with extreme caution. The trial-level data violate the independence assumption required for inferential statistics, as multiple trials from the same participant are correlated. The sample size (N = 20 participants) provides insufficient between-subject degrees of freedom to examine speed × load interaction effects. These data are presented solely to motivate future research employing: (a) independent observations at each speed condition, (b) larger samples allowing between-subject comparisons, and (c) pre-registered hypotheses about speed-dependent MOT–performance relationships.

# S2. Supplementary Descriptive Statistics

**Table S1.** Full correlation matrix (Pearson r) for all study variables, complementing Figure 3 in the main manuscript.

|  | **MOT-2** | **MOT-3** | **MOT-4** | **MOT-5** | **MOT-6** | **MOT-10°/s** | **MOT-15°/s** | **Age** | **Train** | **Score** |
| --- | --- | --- | --- | --- | --- | --- | --- | --- | --- | --- |
| MOT-2 | 1 |  |  |  |  |  |  |  |  | −.208 |
| MOT-3 | .72 | 1 |  |  |  |  |  |  |  | +.039 |
| MOT-4 | .58 | .65 | 1 |  |  |  |  |  |  | −.227 |
| MOT-5 | .45 | .52 | .60 | 1 |  |  |  |  |  | +.132 |
| MOT-6 | .38 | .44 | .50 | .68 | 1 |  |  |  |  | −.106 |
| MOT-10°/s | .35 | .40 | .42 | .38 | .32 | 1 |  |  |  | −.059 |
| MOT-15°/s | .30 | .35 | .38 | .42 | .40 | .55 | 1 |  |  | +.327 |
| Age | .05 | .10 | .08 | .12 | .08 | .06 | .15 | 1 |  | +.741 |
| Train | .08 | .12 | .10 | .15 | .12 | .08 | .18 | .928 | 1 | +.650 |
| Score | −.208 | +.039 | −.227 | +.132 | −.106 | −.059 | +.327 | +.741 | +.650 | 1 |

Note. N = 20. MOT-2 through MOT-6: standard speed (5°/s). MOT-10°/s, MOT-15°/s: increased speeds (10 and 15°/s). Train = Training Years. Lower triangle: Pearson r. Upper triangle omitted for clarity. Values are rounded to two or three decimals as reported in the main manuscript.
